# Integrated polyetheretherketone patient-specific implants for multi-subunit midface concavity: a retrospective case series

**DOI:** 10.3389/fsurg.2026.1868881

**Published:** 2026-07-07

**Authors:** Xin Wang, Sihan Wu, Menghao Wang, Tianhang Wu, Xiangyu Zheng, Fei Li, Xiaowei Wang, Zhihan Hu, Xinyi Chen, Qiming Zhao, Xiaoping Chen, Yue Chen

**Affiliations:** 1 Department of Plastic and Cosmetic Surgery, Zhejiang Hospital, Hangzhou, China; 2 Department of Plastic and Cosmetic Surgery, Hangzhou United Lige Sixth Medical Aesthetic Hospital, Hangzhou, China; 3 Faculty of Medicine, Complutense University of Madrid, Madrid, Spain

**Keywords:** aesthetic surgery, computer-aided design and manufacturing, facial skeletal augmentation, midface concavity, patient-specific implant, polyetheretherketone, reconstructive surgery

## Abstract

**Background:**

Correction of multi-subunit midface concavity in patients without malocclusion remains challenging when multiple prefabricated implants are used, because implant transitions may be irregular and dissection around the infraorbital foramen is technically demanding. This study evaluated the feasibility, safety, and short-term aesthetic outcomes of integrated polyetheretherketone patient-specific implants designed using computer-aided design and manufacturing technology.

**Methods:**

This retrospective case series screened consecutive adults treated with bilateral integrated PEEK patient-specific implants for multi-subunit midface concavity. Eligible patients had normal or essentially normal occlusion, clinical and CT-based moderate-to-severe concavity involving at least two adjacent subunits, adequate soft-tissue coverage, and completed postoperative follow-up. Demographic, implant-planning, satisfaction, and adverse-event data were reviewed. The primary outcome was late postoperative patient-reported aesthetic satisfaction assessed using a modified GAIS-derived 5-point scale. Early (10–30 days) and late (>90 days) satisfaction scores were compared using the Wilcoxon signed-rank test.

**Results:**

Sixty-two implants were placed in 31 patients. The mean follow-up was 8.3 ± 2.1 months. Implant placement was successful in all cases, and no intraoperative reshaping was required. Late postoperative satisfaction scores were significantly higher than early postoperative scores (*P* < 0.01). At late follow-up, 28 patients (90.3%) were satisfied or very satisfied. Transient postoperative edema occurred in all patients, and transient upper-lip or infraorbital-region hypoesthesia occurred in 12 patients (38.7%), resolving spontaneously within 3 months. No infection, implant exposure, migration, extrusion, chronic inflammation, sinus tract formation, or permanent infraorbital nerve injury was observed.

**Conclusion:**

Integrated polyetheretherketone patient-specific implants appear to be a feasible option for selected patients with multi-subunit midface concavity and normal or essentially normal occlusion. The integrated patient-specific design may support customized skeletal augmentation across adjacent midfacial subunits and individualized aesthetic planning. Further studies with longer follow-up, comparative controls, and objective three-dimensional outcome assessment are warranted.

## Introduction

Midface concavity may result from developmental underprojection of the midfacial skeleton or age-related bone resorption and can compromise facial balance and aesthetics (1–3). In patients with malocclusion, correction usually requires orthognathic surgery to address both functional and skeletal abnormalities (4–6). In contrast, patients without clinically significant malocclusion may benefit from isolated skeletal augmentation of the midface rather than orthodontic or orthognathic correction. Alloplastic implants can provide structural support, elevate the overlying soft tissues, and restore midfacial convexity and cheek fullness (7–11).

The midface has been divided into six anatomical subunits: the lower paranasal region, upper paranasal region, superior infraorbital region, inferior infraorbital region, zygomatic region, and subzygomatic region (12). Although lower paranasal deficiency is common, many patients present with concavity involving multiple adjacent subunits rather than an isolated nasal-base defect. Conventional preformed implants, including silicone, porous polyethylene, and expanded polytetrafluoroethylene, are generally designed for specific regions, such as the nasal base, infraorbital rim, or zygomatic–malar area (7–15). These implants can be effective for localized deficiencies, but their combined use in multi-subunit deformities may result in contour discontinuity, visible step-offs, or an irregular soft-tissue surface. In addition, placement of multiple implants around the infraorbital region increases the technical demand of avoiding injury to the infraorbital nerve.

Computer-aided design/computer-aided manufacturing (CAD/CAM) technology has enabled the fabrication of patient-specific implants (PSIs) that conform to complex craniofacial anatomy. Polyetheretherketone (PEEK) is a high-performance polymer increasingly used for craniofacial and facial patient-specific implants because of its favorable mechanical properties, biocompatibility, radiolucency, and processability (16–24). Previous reports have described the use of PEEK patient-specific implants for aesthetic correction of midface concavity, particularly in the paranasal region (25–27). However, published designs have largely focused on localized augmentation rather than integrated reconstruction across multiple midfacial subunits.

To address this clinical gap, we retrospectively reviewed our initial clinical experience with integrated polyetheretherketone patient-specific implants (IPPSIs) for correcting multi-subunit midface concavity in selected patients without clinically significant malocclusion. We aimed to evaluate the feasibility, patient-reported aesthetic outcomes, and short-term safety of this approach.

## Materials and methods

### Study design and ethics

This retrospective case series was designed and reported in accordance with the Strengthening the Reporting of Observational Studies in Epidemiology (STROBE) guidelines. The study was conducted in accordance with the Declaration of Helsinki and was approved by the Institutional Review Board of Zhejiang Hospital, School of Medicine, Zhejiang University (approval number: ZJHIRB-2025-258 K). Written informed consent was obtained from all patients before surgery.

### Patients

**Figure 1:**
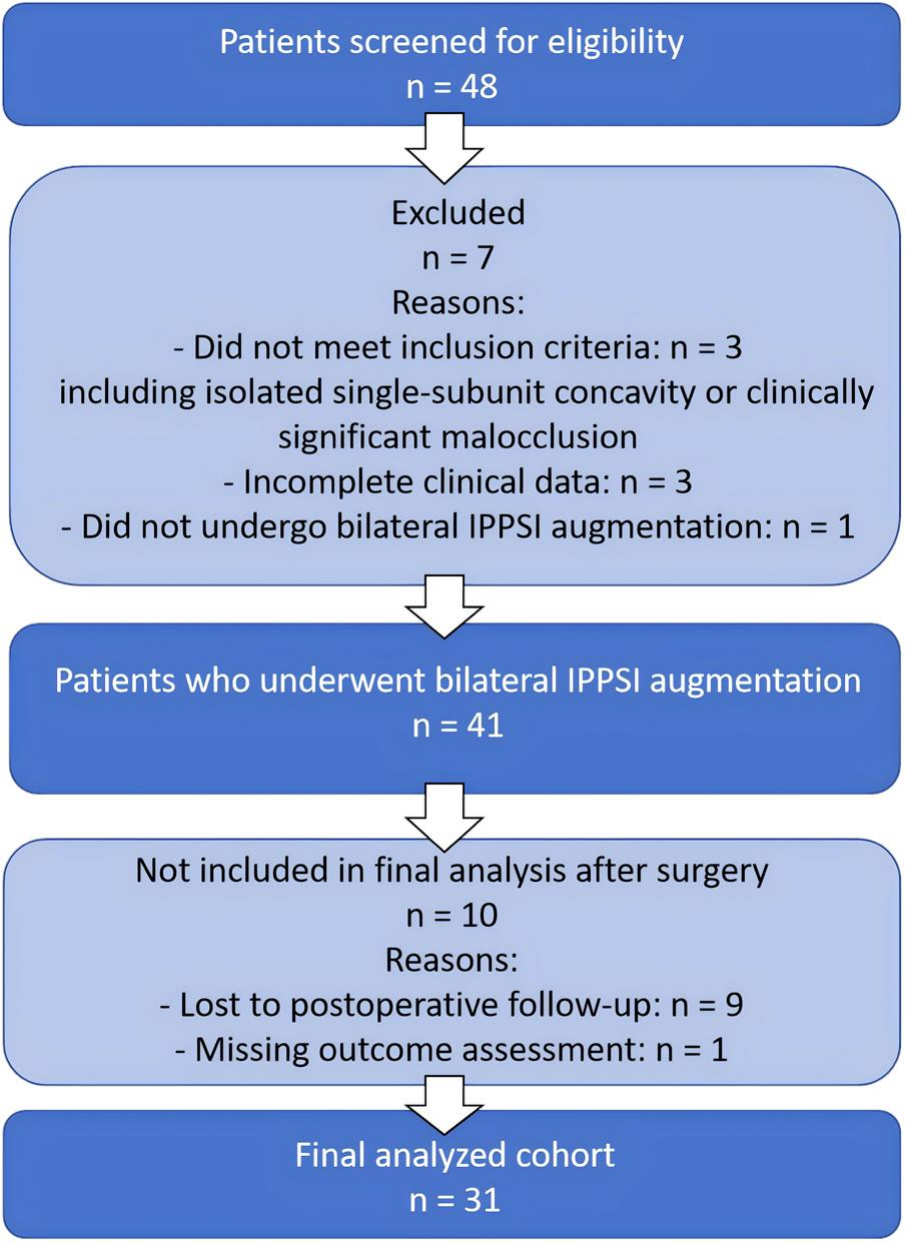
STROBE-style patient flow diagram.

We retrospectively screened consecutive patients who underwent bilateral integrated polyetheretherketone patient-specific implant (IPPSI) augmentation for multi-subunit midface concavity at the Department of Plastic Surgery, Zhejiang Hospital, School of Medicine, Zhejiang University. Clinical examination, standardized preoperative photographs, and maxillofacial CT were used to assess skeletal projection, soft-tissue envelope, and the severity and distribution of midfacial concavity. The flow diagram shows the number of patients screened for eligibility, excluded, lost to follow-up, and included in the final analysis (Figure 1).

Patients were eligible if they met the following criteria: (1) age ≥18 years; (2) normal or essentially normal occlusion; (3) clinical and CT-based evidence of moderate-to-severe midface concavity involving at least two adjacent subunits; (4) adequate soft-tissue coverage without severe scarring, contracture, or active inflammation; and (5) patient request for skeletal contour augmentation using a customized implant.

Normal occlusion was defined as an Angle Class I first molar relationship, well-aligned dentition without obvious crowding, rotation, or malposition, and coincidence of the upper and lower dental midlines, which were approximately aligned with the facial midline. Essentially normal occlusion was defined as an Angle Class I first molar relationship with mild dental crowding not exceeding 3 mm and dental midline deviation within 3 mm. Patients with functional occlusal disturbance or clinically significant maxillomandibular discrepancy requiring orthodontic or orthognathic treatment were excluded.

**Figure 2:**
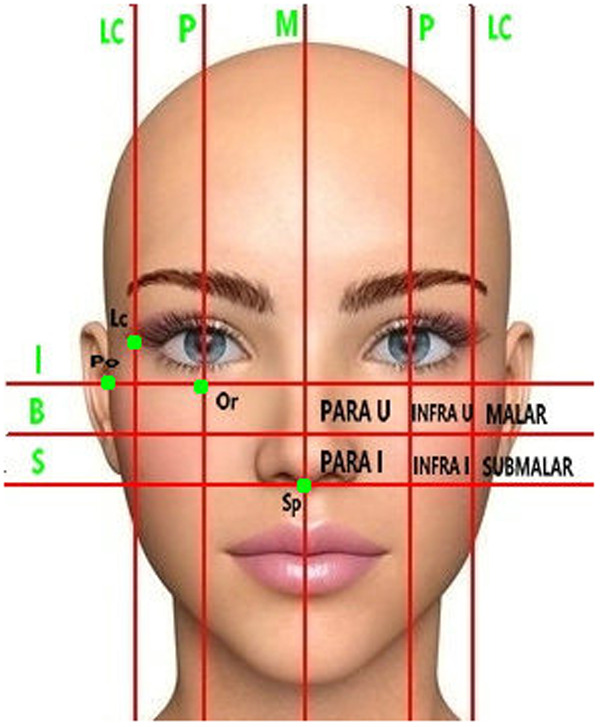
Anatomical subdivision of the midface. PARA-U, upper paranasal; PARA-I, lower paranasal; INFRA-U, superior infraorbital; INFRA-I, inferior infraorbital; MALAR, zygomatic; SUBMALAR, subzygomatic.

The severity of midface concavity was classified according to the anatomical extent of CT-confirmed skeletal deficiency. The midface was divided into six anatomical subunits: the lower paranasal region, upper paranasal region, superior infraorbital region, inferior infraorbital region, zygomatic region, and subzygomatic region (Figure 2). Mild concavity was defined as skeletal deficiency limited to the paranasal region. Moderate concavity was defined as deficiency extending from the paranasal region to the infraorbital region. Severe concavity was defined as deficiency extending further to the zygomatic region and/or zygomatic arch. Therefore, moderate-to-severe multi-subunit midface concavity referred to CT-confirmed skeletal underprojection involving at least two adjacent midfacial subunits beyond an isolated paranasal deficiency.

Patients were excluded if they had Angle Class II or III malocclusion, functional occlusal disturbance, clinically significant maxillary skeletal deformity requiring orthodontic or orthognathic treatment, isolated single-subunit concavity suitable for localized augmentation, active oral, sinus, or facial infection, a history of midfacial tumor resection, major trauma or severe scarring substantially altering normal anatomy, insufficient preoperative CT data, or inability or unwillingness to complete postoperative follow-up.

Patients were classified as primary or secondary/revision cases according to previous midfacial surgical history. Primary cases were defined as patients without previous skeletal midface contouring or midfacial implant surgery, whereas secondary/revision cases were defined as those with previous midfacial contouring procedures, including prior zygomatic reduction or zygomaticoplasty.

### Sample size

Because this was a retrospective exploratory case series describing an early clinical experience with integrated PEEK patient-specific implants, no *a priori* sample size calculation was performed. The sample size was determined by the number of consecutive eligible patients treated during the study period who met the inclusion and exclusion criteria and completed postoperative follow-up.

### Indication and subunit-specific planning

For patients who met the above eligibility criteria, subunit-specific augmentation was planned using a combination of clinical facial analysis, standardized photographs, CT-based skeletal assessment, and patient-specific aesthetic concerns. CT-based three-dimensional skull modeling was used to identify skeletal underprojection in each involved midfacial subunit, whereas clinical examination was used to evaluate the visible contour deficiency, soft-tissue envelope, and aesthetic areas requiring correction.

The extent of implant coverage was individualized according to the distribution of CT-identified skeletal deficiency, soft-tissue adequacy, and the patient's aesthetic goals. In general, an integrated implant was planned when augmentation of two or more adjacent midfacial subunits was required to restore a continuous midfacial contour. Therefore, implant coverage was patient-specific rather than based on a fixed template or rigid algorithm. The CT-identified deficient subunits and planned implant-covered subunits for each patient are summarized in Supplementary Table S1.

### Implant design and fabrication

**Figure 3:**
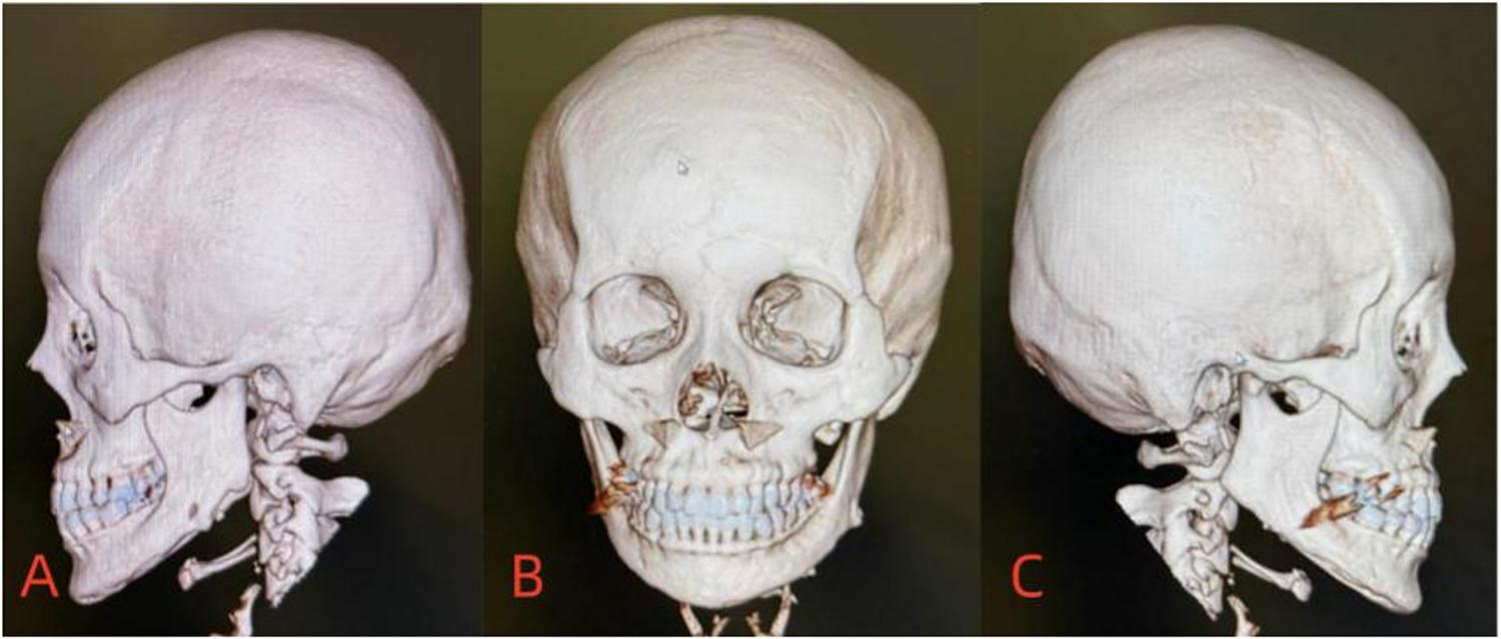
Preoperative CT-based craniofacial reconstruction showing the extent of midface concavity and any pre-existing implants. **(A)** Left oblique view. **(B)** Frontal view. **(C)** Right oblique view.

**Figure 4:**
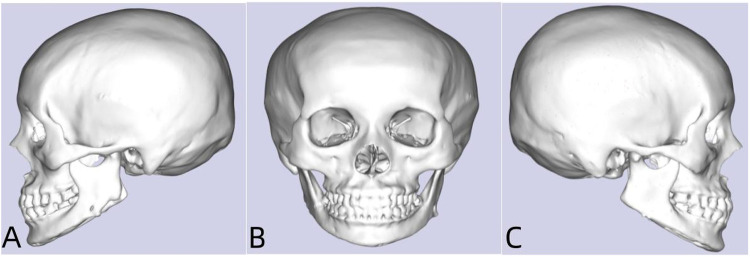
Virtual 3D skull model illustrating the distribution of deficient midfacial subunits. **(A)** Left oblique view. **(B)** Frontal view. **(C)** Right oblique view.

All patients underwent high-resolution maxillofacial computed tomography with a slice thickness of 1 mm (Figure 3). The DICOM data were imported into medical image-processing software (Mimics, version 19.0; Materialise NV, Leuven, Belgium) to reconstruct the craniofacial skeleton and assess the size and morphology of skeletal deficiency in each affected midfacial subunit (Figure 4). Previously implanted materials, such as silicone or expanded polytetrafluoroethylene, were also identified when present.

**Figure 5:**
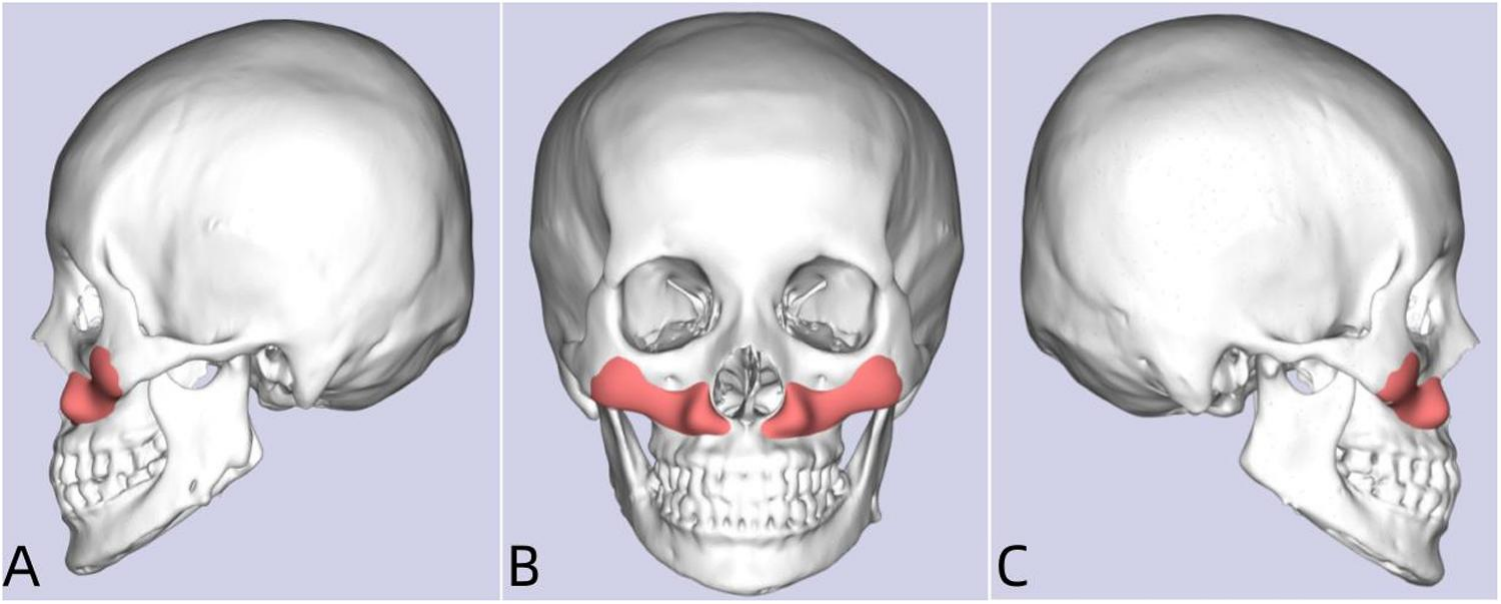
Preoperative virtual design of the integrated patient-specific implant. **(A)** Left oblique view. **(B)** Frontal view. **(C)** Right oblique view.

Imaging and design data were processed in collaboration with the PSI manufacturer, Xi’an Kangtuo Medical Technology Co., Ltd. (Xi’an, China), as part of the routine CAD/CAM production workflow. The reconstructed three-dimensional skull model was transferred to 3D design software (3-matic, version 19.0; Materialise NV, Leuven, Belgium) for implant planning. For each patient, CT-identified deficient subunits and planned implant-covered subunits were recorded separately to distinguish anatomical deficiency from individualized aesthetic planning; these patient-level design characteristics are summarized in Supplementary Table S1. When anatomically appropriate, one side of the implant was designed using surface-based modeling, and the contralateral implant was generated using mirror imaging to improve bilateral symmetry (Figure 5).

Implant location, boundary, contour, thickness, and fixation sites were jointly determined by the surgeon and biomedical engineers according to skeletal deficiency, soft-tissue envelope, patient-specific aesthetic requirements, and the anatomical position of the infraorbital foramen. The proposed design was reviewed with the patient before final fabrication. Although the final geometry was individualized, all cases followed the same CAD/CAM workflow: CT-based skeletal reconstruction, deficient-subunit mapping, virtual augmentation design, edge tapering, infraorbital foramen avoidance, screw-hole planning, prototype verification, and final surgical approval before fabrication.

During virtual design, the infraorbital foramen was identified on the CT-based three-dimensional skull model. An avoidance area was incorporated around the foramen to prevent direct implant compression of the infraorbital neurovascular bundle. The implant margin and screw-hole positions were adjusted accordingly, and screw holes were planned in stable bony areas, usually in the anterior maxillary, paranasal, or zygomatic buttress regions, away from the infraorbital foramen.

**Figure 6:**
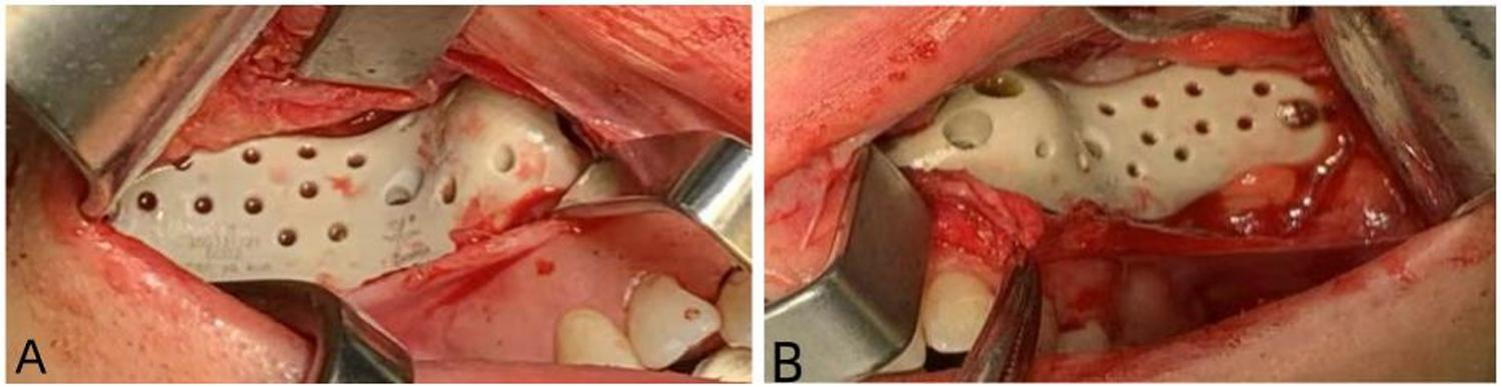
Intraoperative placement and screw fixation of the IPPSI. **(A)** Right side. **(B)** Left side.

To improve reproducibility, key design parameters were reviewed during CAD planning. In this series, the maximal augmentation projection was generally planned within approximately 3–8 mm, the central implant thickness was usually 2–5 mm, and the peripheral margin was tapered to approximately 0.5–1.0 mm to reduce palpability and visible step-off deformity. The estimated implant volume generally ranged from approximately 2–8 cm^3^ per side, depending on the number of covered subunits and the planned degree of augmentation. Screw-fixation areas were designed with sufficient thickness for stable fixation. Each implant was usually fixed with two to three titanium screws, 2.0 mm in diameter and 6–8 mm in length (Figure 6). The milled implant surface was smooth, without porous coating or additional surface modification in this series.

**Figure 7:**
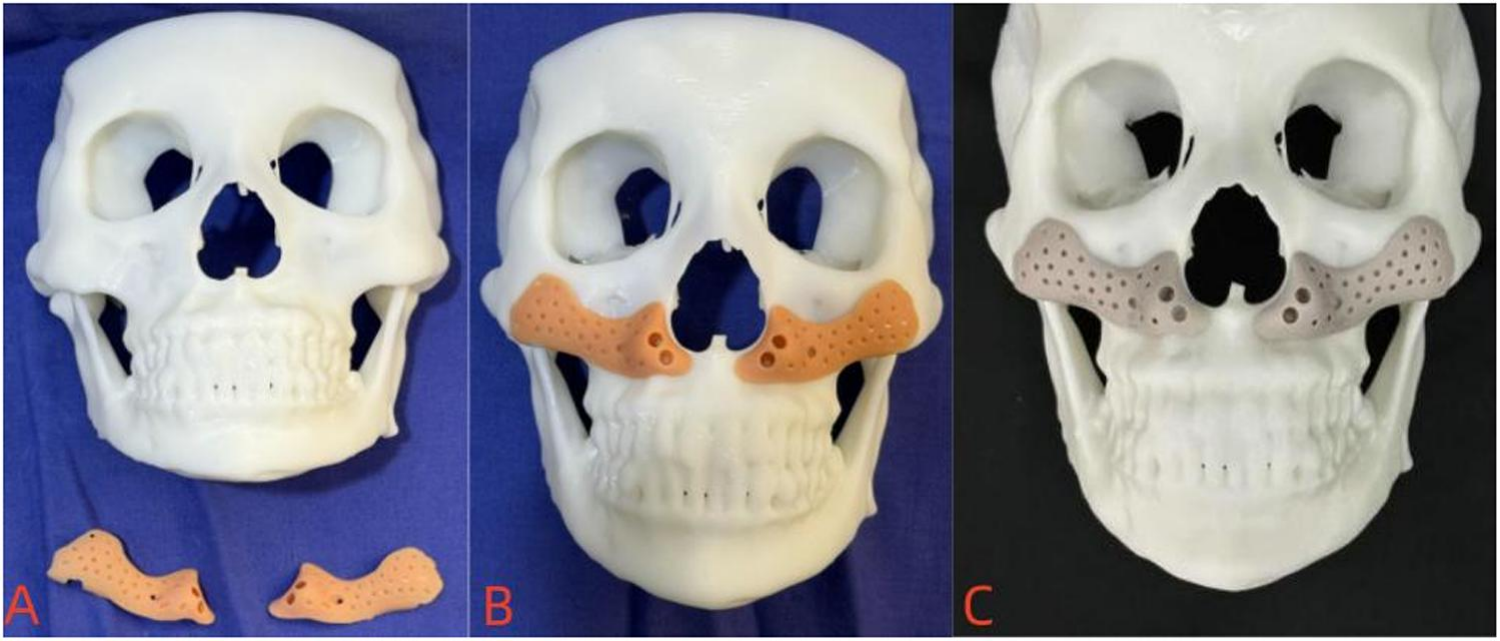
Design verification using three-dimensional printed models. **(A)** Three-dimensional printed skull and implant prototypes. **(B)** Simulated implant placement on the skull model for contour assessment. **(C)** Final IPPSI manufactured from polyetheretherketone.

The approved virtual design was converted into stereolithography format, and a patient-specific skull model with a prototype implant was fabricated using three-dimensional printing. The prototype implant was placed on the printed skull model to allow direct visual and manual assessment of the anticipated skeletal contour by both the surgeon and the patient (Figures 7A,B). After patient confirmation and final surgeon approval, the definitive IPPSI was manufactured from medical-grade PEEK using computer numerical control milling, a subtractive manufacturing technique, and sterilized preoperatively according to standard protocol (Figure 7C).

### Surgical procedure

All procedures were performed as routine clinical implant placement through an intraoral upper vestibular incision. A subperiosteal pocket was created according to the preoperative virtual design, and the IPPSI was inserted, seated on the recipient bone surface, and fixed with titanium screws. The incision was closed in layers, and postoperative care followed the standard institutional protocol.

### Outcome assessment

The primary outcome was patient-reported aesthetic satisfaction at the late postoperative assessment. Secondary outcomes included early postoperative satisfaction, change in satisfaction between early and late assessments, and postoperative safety findings.

Aesthetic satisfaction was the primary outcome of this study and reflected patients’ subjective satisfaction with the cosmetic result of surgery. The Global Aesthetic Improvement Scale (GAIS) is a validated generic assessment scale that has been widely used in aesthetic medicine and facial aesthetic studies to monitor aesthetic improvement and treatment outcomes. In the present study, aesthetic satisfaction was evaluated using a modified GAIS-based 5-point patient-reported satisfaction scale: 5, Very satisfied; 4, Satisfied; 3, Slight improvement; 2, Dissatisfied; and 1, Very dissatisfied. This modified GAIS-derived satisfaction scale was used as a pragmatic patient-reported assessment tool in routine follow-up. No independent blinded aesthetic assessment or objective three-dimensional postoperative morphometric analysis was performed.

Early postoperative satisfaction was defined as the first available satisfaction assessment performed within 10–30 days after surgery. Satisfaction was not assessed immediately after surgery because patients wore a dedicated postoperative head dressing and could not adequately observe their facial contour. The earliest assessment was generally performed after suture removal at approximately 10 days. If obvious ecchymosis or bruising was present, assessment was delayed until these findings had improved.

Clinical safety was assessed throughout the perioperative and follow-up periods. Intraoperative events, wound healing problems, sensory changes, implant-related adverse events, revision surgery, and implant removal were recorded based on operative reports, clinical examinations, follow-up records, and imaging findings. Infraorbital nerve–related sensory disturbance was assessed during routine postoperative follow-up based on clinical examination and patient-reported symptoms, including numbness, paresthesia, or altered sensation in the infraorbital nerve distribution. The presence and resolution of sensory symptoms were recorded when documented in the medical records. Persistent sensory deficit at the last follow-up was classified as an unresolved sensory complication.

## Statistical analysis

Statistical analysis was performed using SPSS software, version 30.0. Continuous variables are presented as mean ± standard deviation or median with interquartile range (IQR), as appropriate. Categorical variables are presented as numbers and percentages. Because patient-reported satisfaction scores were ordinal data, paired early and late postoperative satisfaction scores were compared using the Wilcoxon signed-rank test. The 95% confidence intervals for proportions were calculated using the Wilson score method. A two-sided *P* value < 0.05 was considered statistically significant.

## Results

Patient demographic and clinical characteristics are summarized in Table 1. Thirty-one patients with multi-subunit midface concavity underwent CAD/CAM-customized IPPSI augmentation. The cohort included 6 men and 25 women, with a mean age of 29.00 years and an age range of 20–50 years. A total of 62 IPPSIs were implanted. The mean follow-up duration was 8.3 ± 2.1 months, with a range of 6–12 months. Eight patients (25.8%) underwent surgery under general anesthesia, and 23 patients (74.2%) underwent surgery under local anesthesia. The most frequently CT-identified deficient subunits were the lower paranasal region (30/31, 96.77%), inferior infraorbital region (29/31, 93.55%), and zygomatic/malar region (25/31, 80.65%). Detailed patient-level data, including CT-identified deficient subunits, implant-covered subunits, CT-based skull modeling, and virtual implant designs, are provided in Supplementary Table S1 to illustrate the individualized planning process. Regarding previous surgical history, 30 patients (96.77%) were primary cases without prior midfacial skeletal contouring surgery, whereas one patient (3.23%) was classified as a secondary/revision case because of previous reduction malarplasty and zygomatic archplasty.

**Table 1:** Demographic and clinical characteristics of the patients.

Variable	Value
Demographic and clinical characteristics	
Patients, n	31
Implants, n	62
Age, years, mean ± SD	29.00 ± 7.22
Age range, years	20–50
Male, *n* (%)	6 (19.35%)
Female, *n* (%)	25 (80.65%)
Follow-up duration, months, mean ± SD	8.3 ± 2.1
Follow-up range, months	6–12
Bilateral implantation, *n* (%)	31 (100.00%)
Anesthesia modality	
General anesthesia, *n* (%)	8 (25.81%)
Local anesthesia, *n* (%)	23 (74.19%)
CT-identified deficient subunits	
Lower paranasal region, *n* (%)	30 (96.77%)
Inferior infraorbital region, *n* (%)	29 (93.55%)
Zygomatic/malar region, *n* (%)	25 (80.65%)
Superior infraorbital region, *n* (%)	22 (70.97%)
Upper paranasal region, *n* (%)	16 (51.61%)
Subzygomatic/submalar region, *n* (%)	2 (6.45%)
Implant-covered subunits	
Lower paranasal region, *n* (%)	30 (96.77%)
Inferior infraorbital region, *n* (%)	27 (87.10%)
Upper paranasal region, *n* (%)	16 (51.61%)
Zygomatic/malar region, *n* (%)	18 (58.06%)
Superior infraorbital region, *n* (%)	11 (35.48%)
Surgical history	
Primary cases, *n* (%)	30 (96.77%)
Secondary/revision cases, *n* (%)	1 (3.23%)
Previous reduction malarplasty and zygomatic archplasty, *n* (%)	1 (3.23%)

CT, computed tomography; SD, standard deviation.

## Patient-reported satisfaction

Patient-reported satisfaction was assessed using a modified 5-point GAIS-derived scale at an early postoperative time point during the edematous phase and at a later postoperative time point after edema resolution. In the early postoperative assessment, 26 patients rated their outcome as “Slight improvement” and 5 patients as “Satisfied.” No patients reported being “Very satisfied,” “Dissatisfied,” or “Very dissatisfied” at this stage.

At the late postoperative assessment, patient-reported satisfaction scores were higher than those at the early assessment using the Wilcoxon signed-rank test (*P* < 0.01; Table 2). Eighteen patients reported being “Very satisfied,” 10 were “Satisfied,” 1 reported “Slight improvement,” and 2 were “Dissatisfied.” The proportion of patients reporting “Very satisfied” or “Satisfied” was 90.32% (28/31).

**Table 2:** Patient-reported satisfaction at early and late postoperative assessments.

Score	Grade	Early postoperative period (10–30D)	Late postoperative period (>90D)	*P* value
1	Very dissatisfied	0 (0.00%)	0 (0.00%)	
2	Dissatisfied	0 (0.00%)	2 (6.45%)	
3	Slight improvement	26 (83.87%)	1 (3.23%)	
4	Satisfied	5 (16.13%)	10 (32.26%)	
5	Very satisfied	0 (0.00%)	18 (58.06%)	
Overall score, median (IQR)		3 (3-3)	5 (4-5)	<0.01

Data are presented as *n* (%) or median (IQR). Statistical comparison of paired early and late postoperative satisfaction scores was performed using the Wilcoxon signed-rank test. IQR, interquartile range.

Among the two dissatisfied patients, one experienced mild upper-lip skin irregularity during forceful smiling, which resolved spontaneously within 6 months. The other patient perceived the implant as oversized despite no obvious abnormality on clinical examination and subsequently underwent implant removal at another institution.

## Surgical safety and adverse events

Postoperative findings and adverse events are summarized in Table 3. Transient postoperative edema was observed in all patients (31/31, 100.0%; 95% CI, 88.8–100.0%), typically peaking on postoperative days 3–4 and gradually resolving within 1–3 months. Transient upper-lip or infraorbital-region hypoesthesia occurred in 12 patients (38.7%; 95% CI, 21.8–57.8%) and resolved spontaneously within 3 months. Two patients (6.5%; 95% CI, 0.8–21.4%) reported temporary foreign-body sensation or stiff smiling. One patient (3.2%; 95% CI, 0.1–16.7%) underwent implant removal at an external institution because of subjective dissatisfaction with implant size or perceived overcorrection. No surgical site infection, hematoma requiring intervention, implant exposure, extrusion, migration or displacement, chronic inflammation, sinus tract formation, or permanent infraorbital nerve injury was observed during follow-up.

**Table 3:** Postoperative findings and adverse events.

Category	Finding/adverse event	Patients, n/N (%)	95% CI	Management	Outcome
Expected postoperative finding	Transient postoperative edema	31/31 (100.0%)	88.8–100.0%	Routine postoperative care	Resolved gradually within 1–3 months
Minor adverse event	Transient upper-lip or infraorbital-region hypoesthesia	12/31 (38.7%)	21.8–57.8%	Conservative observation	Resolved spontaneously within 3 months
Minor adverse event	Temporary foreign-body sensation or stiff smiling	2/31 (6.5%)	0.8–21.4%	Observation and reassurance	Resolved in one patient; mild symptom persisted in one patient at 3 months
Revision event	Subjective dissatisfaction with implant size/perceived oversizing	1/31 (3.2%)	0.1–16.7%	Implant removal at an external institution	No infection, exposure, or displacement was reported
Major implant-related complication	Infection, exposure, extrusion, displacement, chronic inflammation, sinus tract formation, or permanent infraorbital nerve injury	0/31 (0.0%)	0.0–11.2%	Not applicable	Not observed

The 95% CIs were calculated using the Wilson score method. Events were reported at the patient level unless otherwise specified. Major implant-related complications included surgical site infection, hematoma requiring intervention, implant exposure, extrusion, migration or displacement, chronic inflammation, sinus tract formation, and permanent infraorbital nerve injury. Implant removal due to aesthetic dissatisfaction was categorized as a revision event rather than a major implant-related complication.

CI, confidence interval; PEEK, polyetheretherketone.

## Representative cases

**Figure 8:**
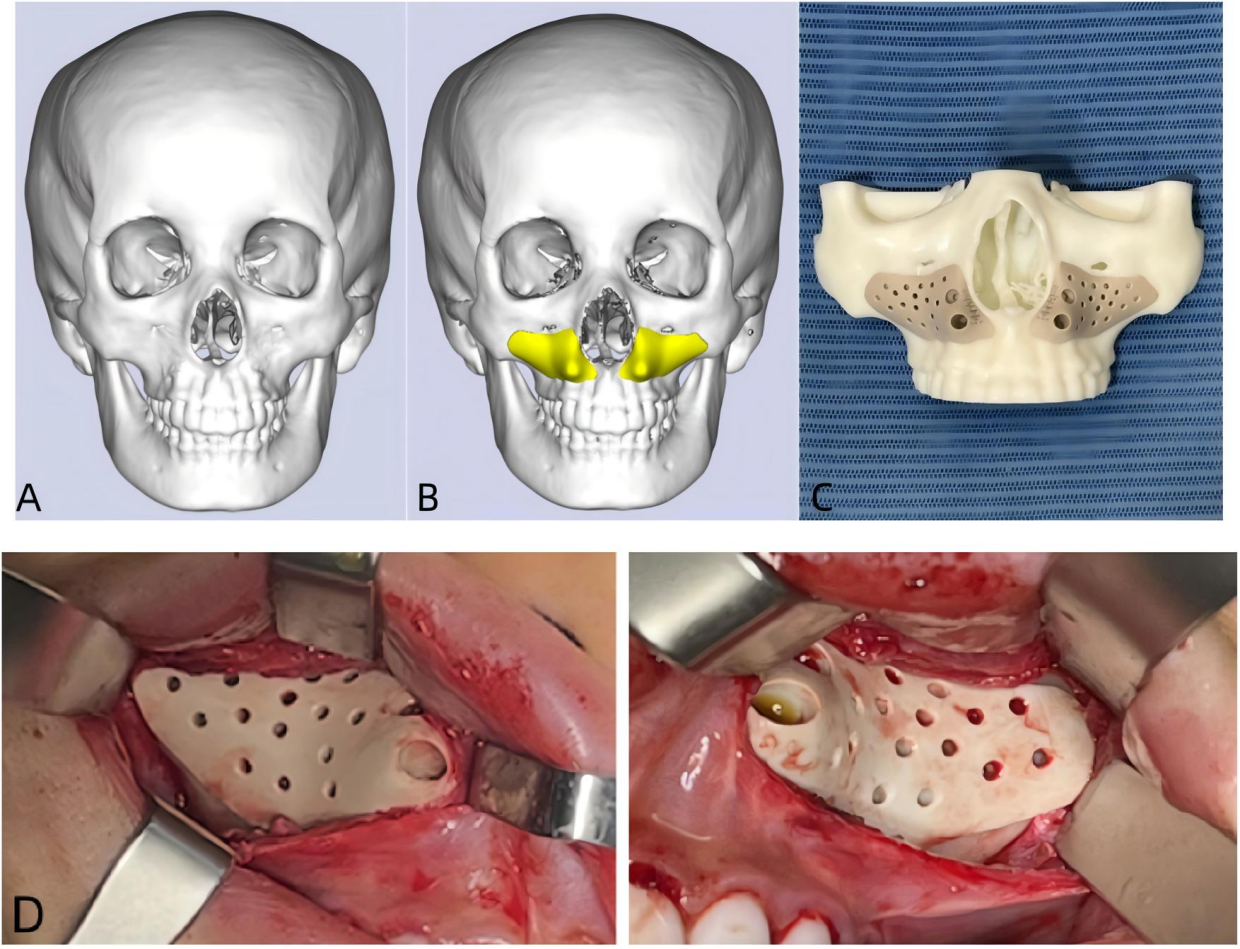
Representative case 1: preoperative defect analysis, virtual design, manufactured implant, and intraoperative fit. **(A)** CT showing PARA-I, INFRA-I, and MALAR deficiency. **(B)** Virtual implant design based on patient goals. **(C)** Final CAD/CAM-manufactured IPPSI. **(D)** Intraoperative placement.

**Figure 9:**
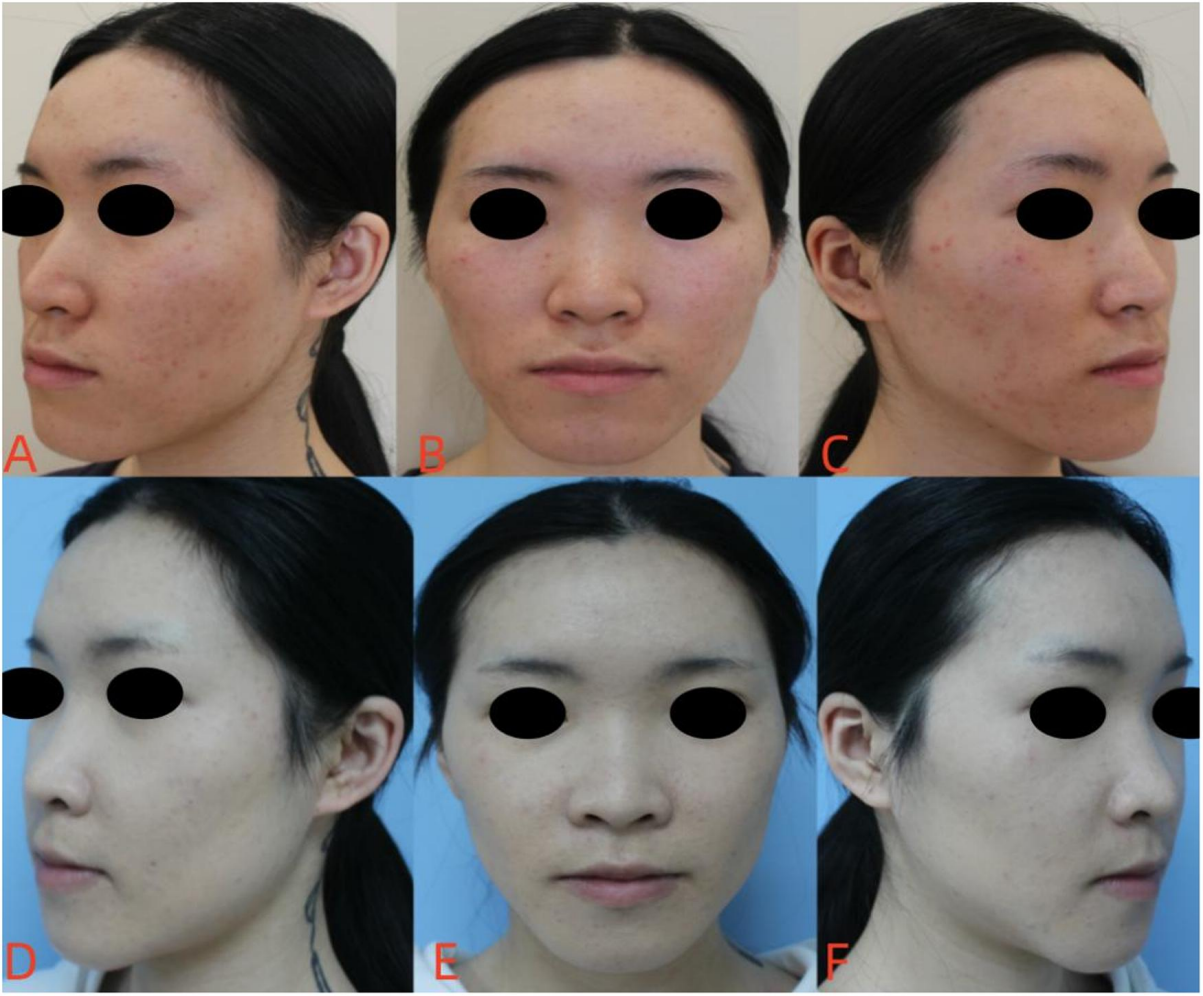
Representative case 1 before and 12 months after surgery. **(A–C)** Preoperative left oblique, frontal, and right oblique views. **(D–F)** Corresponding postoperative views.

**Figure 10:**
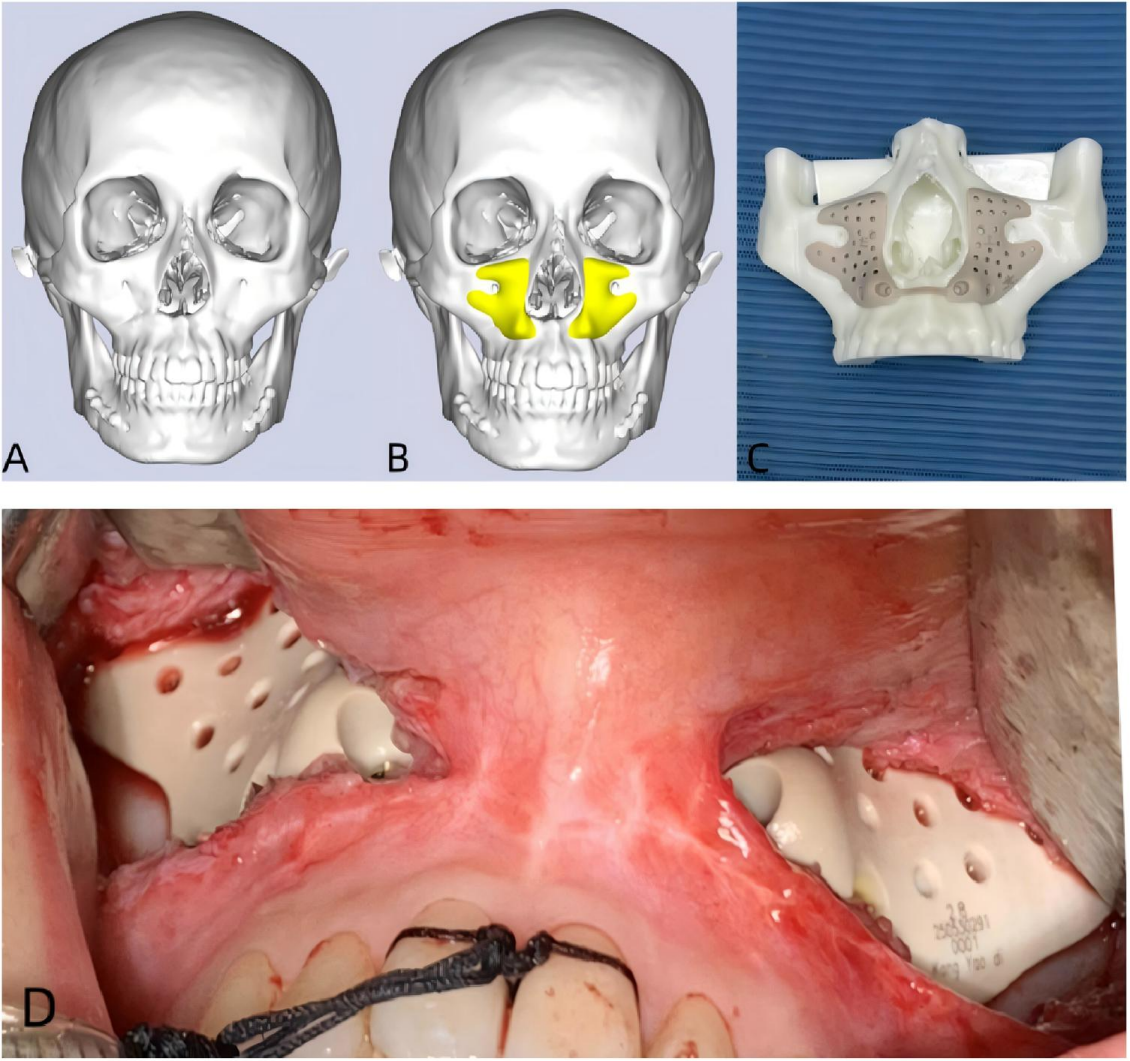
Representative case 2: preoperative defect analysis, virtual design, manufactured implant, and intraoperative fit. **(A)** CT showing PARA-I, PARA-U, INFRA-I, INFRA-U, and MALAR deficiency. **(B)** Virtual implant design based on patient goals. **(C)** Final CAD/CAM-manufactured IPPSI. **(D)** Intraoperative placement.

**Figure 11:**
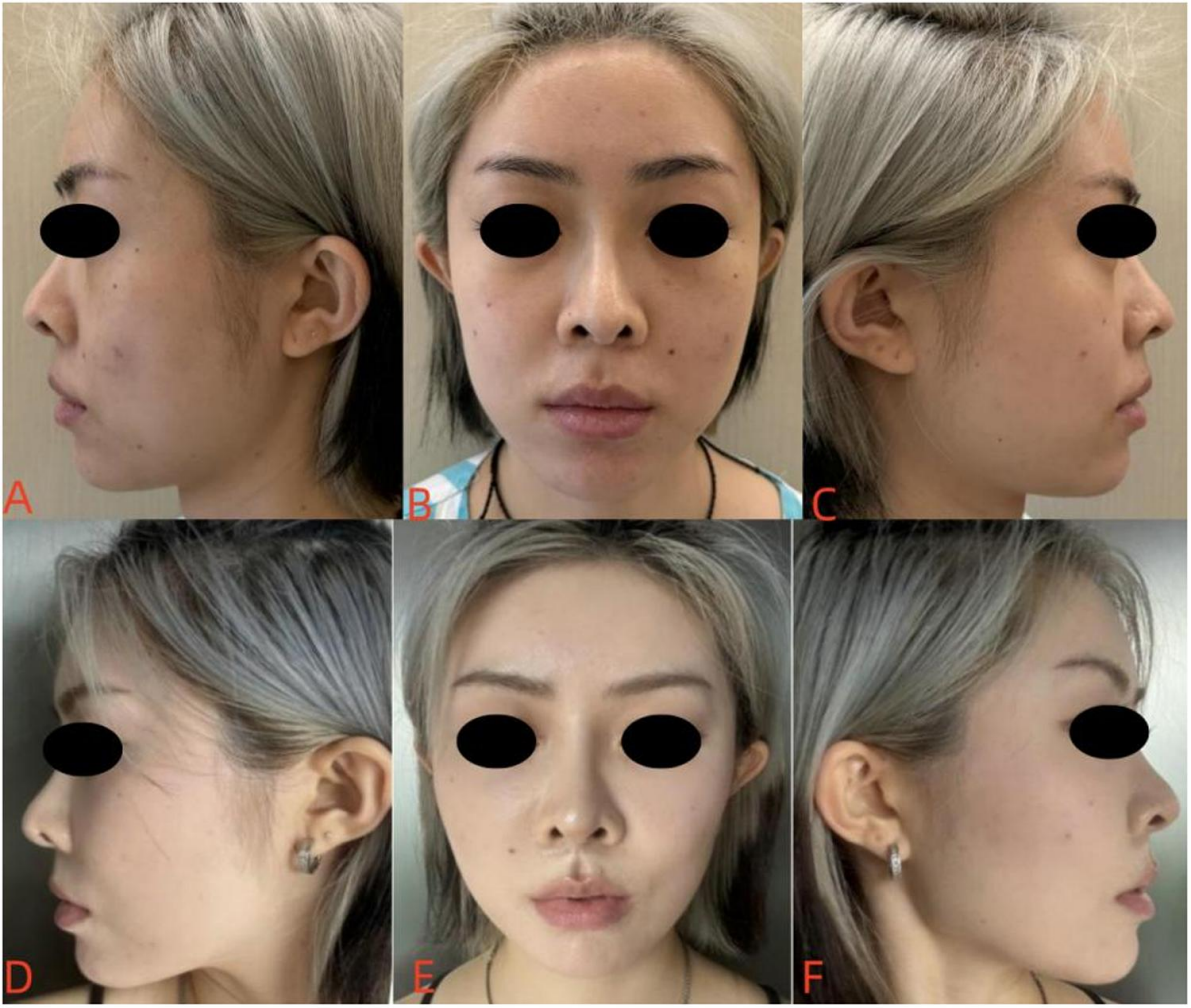
Representative case 2 before and 3 months after surgery. **(A–C)** Preoperative left oblique, frontal, and right oblique views. **(D–F)** Corresponding postoperative views.

**Figure 12:**
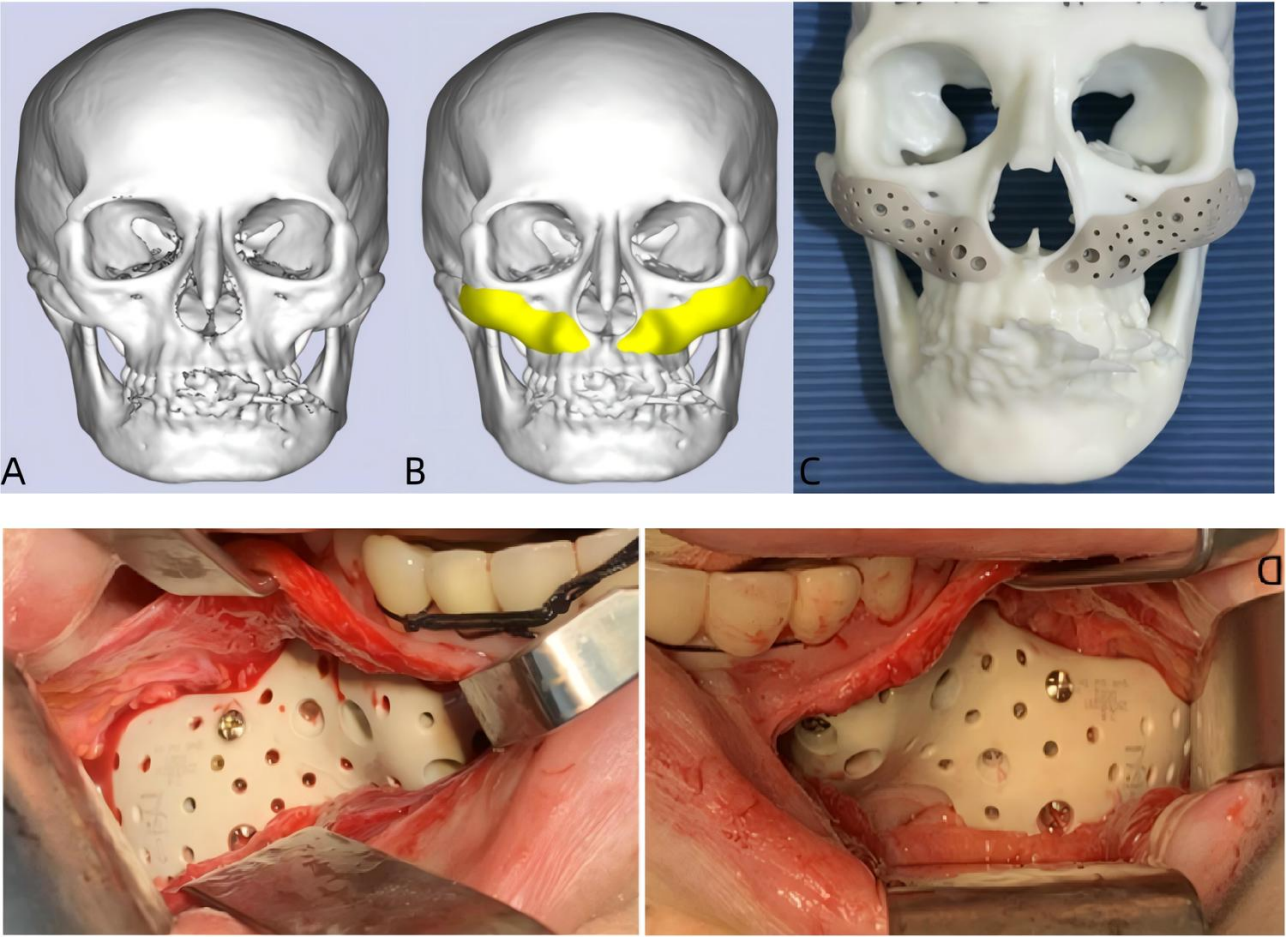
Representative case 3: preoperative defect analysis, virtual design, manufactured implant, and intraoperative fit. **(A)** CT showing PARA-I, INFRA-I, INFRA-U, and MALAR deficiency. **(B)** Virtual implant design based on patient goals. **(C)** Final CAD/CAM-manufactured IPPSI. **(D)** Intraoperative placement.

**Figure 13:**
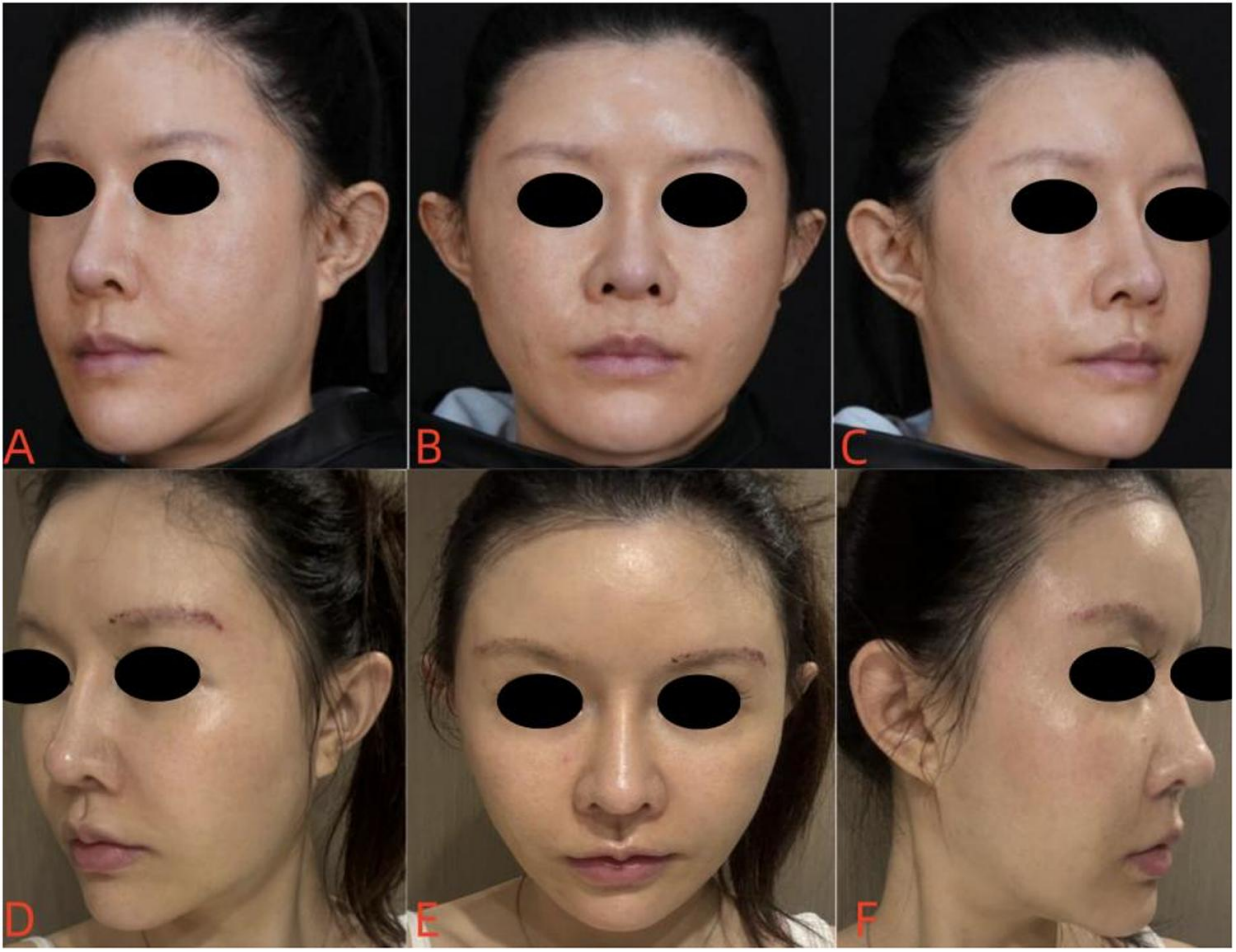
Representative case 3 before and 6 months after surgery. **(A–C)** Preoperative left oblique, frontal, and right oblique views. **(D–F)** Corresponding postoperative views.

Three representative cases are shown in Figures 8–13. Case 1 illustrates an IPPSI designed for the lower paranasal and inferior infraorbital regions in a 23-year-old woman with multi-subunit skeletal deficiency (Figures 8, 9). Case 2 demonstrates correction of upper and lower paranasal and infraorbital concavity in a 30-year-old woman who requested improvement of paranasal depression and a protrusive-lip appearance (Figures 10, 11). Case 3 shows integrated augmentation of the lower paranasal, superior and inferior infraorbital, and malar regions in a 34-year-old woman with midface concavity after previous reduction malarplasty and zygomatic archplasty (Figures 12, 13).

## Discussion

PEEK patient-specific implants have increasingly been used for aesthetic correction of midfacial skeletal deficiency (25–27). However, previous reports have mainly focused on localized augmentation, particularly in the paranasal region, and evidence regarding integrated implant design for simultaneous reconstruction of multiple midfacial subunits remains limited. In this retrospective case series, we used CAD/CAM technology to design integrated PEEK patient-specific implants extending from the paranasal region to the infraorbital and/or malar regions according to each patient's skeletal anatomy and aesthetic goals.

The main advantage of IPPSI is its ability to provide a continuous three-dimensional contour across adjacent midfacial subunits. Conventional prefabricated implants are limited by fixed shapes and sizes, and their use in complex multi-subunit concavity may result in poor adaptation to the maxillary and zygomatic bone surfaces, visible transition lines, or an unnatural soft-tissue contour (11). By contrast, CAD/CAM-based customization allows the implant to be designed according to the patient's CT-derived skeletal morphology. On intraoperative clinical inspection, the implants appeared to fit the planned recipient sites without obvious visible gaps, and no intraoperative implant modification was required.

Compared with conventional alloplastic implants such as silicone and expanded polytetrafluoroethylene (ePTFE), IPPSI may offer advantages in multi-subunit midface augmentation by reconstructing adjacent skeletal regions as a single patient-specific framework (28, 29). Although silicone and ePTFE implants remain effective in selected patients, reported limitations include infection, displacement or malposition, extrusion, bone resorption, contour visibility, palpability, and suboptimal aesthetic outcomes (30, 31). CAD/CAM-designed PEEK implants can be tailored to the patient's skeletal morphology, with tapered margins and planned screw fixation to improve adaptation, contour continuity, and early positional stability (24). In this series, no implant migration, rotation, exposure, or extrusion was observed during short-term follow-up. However, because no direct control group was included and the mean follow-up was only 8.3 months, these findings should be interpreted as preliminary short-term observations rather than evidence of superiority or long-term stability. Comparative studies with longer follow-up are required.

The extent of augmentation should be individualized. Although CT imaging may reveal deficiency in multiple subunits, not all patients desire correction of every affected region. In this series, some patients preferred correction limited to the paranasal and infraorbital regions and declined malar or submalar augmentation to avoid excessive lateral midface widening. Therefore, surgical planning should balance anatomical correction with patient-specific aesthetic expectations. The surgeon should provide professional guidance, but the final implant design should be based on shared decision-making rather than a standardized ideal contour.

Subperiosteal placement provides direct skeletal support and avoids the superficial fullness that may occur with soft-tissue fillers. However, the final soft-tissue elevation is usually smaller than the implant thickness because of the buffering effect of the periosteum, muscle, subcutaneous tissue, and skin. Previous studies reported that postoperative soft-tissue elevation after paranasal augmentation was lower than the actual implant thickness when porous polyethylene or ePTFE implants were used (8, 9). Therefore, mild planned overcorrection may be necessary to achieve clinically visible improvement, particularly in patients with obvious skeletal depression. Nevertheless, excessive implant thickness may increase the risk of foreign-body sensation, unnatural animation, or patient dissatisfaction.

Transient sensory disturbance in the infraorbital nerve territory was the most frequent minor adverse event in this series. Midface augmentation involving the infraorbital, paranasal, malar, or submalar regions carries an inherent risk of infraorbital nerve irritation because the nerve exits through the infraorbital foramen and supplies sensation to the lower eyelid, cheek, lateral nose, and upper lip (32). In the present technique, subperiosteal dissection across multiple adjacent midfacial subunits may expose or manipulate the infraorbital neurovascular bundle. Temporary hypoesthesia is therefore likely related to traction, local edema, compression, or transient neurapraxia rather than direct nerve transection, as described in maxillofacial surgery and midfacial trauma (33). In our cohort, all sensory disturbances resolved spontaneously within 3 months, and no permanent infraorbital nerve injury was observed. These findings emphasize the importance of preoperative localization of the infraorbital foramen, gentle subperiosteal dissection, avoidance of excessive traction, and implant design that prevents direct compression of the infraorbital neurovascular bundle (34).

Postoperative edema was common in this study and represented the main reason for lower satisfaction during the early postoperative period. Edema may temporarily produce pseudo-overcorrection or asymmetry, which can cause patient anxiety. In our cohort, swelling generally peaked within several days and resolved gradually within 1–3 months. Accordingly, patients should be counseled preoperatively that the early postoperative appearance may not reflect the final aesthetic result, and definitive evaluation should be delayed until edema has subsided.

The short-term safety profile of IPPSI appeared acceptable in this initial series. No infection, implant exposure, migration, extrusion, chronic inflammation, sinus tract formation, or permanent infraorbital nerve injury was observed during the available follow-up. Transient hypoesthesia occurred when the infraorbital nerve was exposed during dissection, but all cases resolved spontaneously within three months. One patient underwent implant removal because of perceived oversizing. Previous studies have reported that aesthetic dissatisfaction is one of the most common complications after facial implant surgery (35, 36). These findings suggest that IPPSI can be performed without major implant-related complications in carefully selected patients during short-term follow-up. Nevertheless, the present follow-up duration is insufficient to draw firm conclusions regarding long-term safety, implant stability, or delayed complications.

This study has several limitations. First, this was a single-center retrospective case series with a relatively small sample size, and selection bias could not be completely excluded despite consecutive screening. The absence of a conventional implant control group also precluded direct comparison with standard alloplastic techniques. Second, outcome assessment relied mainly on clinical evaluation, standardized photographs, and a modified 5-point GAIS-based satisfaction scale, rather than validated PROMs, independent assessment, or objective three-dimensional outcome analysis. Third, although CT-based three-dimensional modeling was used for skeletal mapping and implant design, the severity of concavity and extent of implant coverage were determined using an anatomical clinical-radiographic framework rather than validated quantitative cephalometric or morphometric thresholds. Standardized neurosensory testing was also not performed. Finally, the mean follow-up duration was relatively short, and implant position or stability was not assessed using objective postoperative imaging. Therefore, the absence of implant migration, exposure, extrusion, chronic inflammation, or permanent nerve injury should be interpreted as short-term clinical findings only. Larger prospective comparative studies with longer follow-up, validated PROMs, objective three-dimensional assessment, standardized neurosensory testing, and imaging-based evaluation of implant stability are needed.

## Conclusion

Integrated polyetheretherketone patient-specific implants may represent a feasible and individualized option for selected patients with multi-subunit midface concavity and normal or essentially normal occlusion. In this initial retrospective series, the technique was associated with high short-term patient-reported satisfaction and no clinically evident major implant-related complications during follow-up. The integrated patient-specific design provides a practical framework for customized skeletal augmentation across adjacent midfacial subunits. Further prospective comparative studies with validated PROMs, objective three-dimensional assessment, imaging-based evaluation of implant position, and longer follow-up are warranted to confirm its long-term safety, stability, and clinical effectiveness.

## Data Availability

The original contributions presented in the study are included in the article/Supplementary Material, further inquiries can be directed to the corresponding author.
