# Supplementary material for: Integrated polyetheretherketone patient-specific implants for multi-subunit midface concavity: a retrospective case series
Source: Front Surg. 2026 Jul 7;13:1868881. doi: 10.3389/fsurg.2026.1868881 (PMC13385320; doi:10.3389/fsurg.2026.1868881)
Supplement: Supplementary file 1 [file Table1.docx]

**Supplementary Table 1.** Demographic and clinical characteristics of the 31 patients.

| Patient No. | Age, years | Sex | CT-identified deficient subunits | Implant-covered subunits | CT-based skull model | Virtual implant design |
| --- | --- | --- | --- | --- | --- | --- |
| 1 | 20 | Female | PARA I  INFRA I  MALAR | PARA I  INFRA I  MALAR | 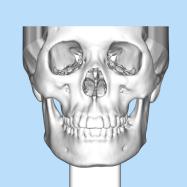 | 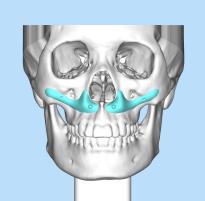 |
| 2 | 25 | Female | PARA I  INFRA U  INFRA I  MALAR  SUBMALAR | PARA I  INFRA I | 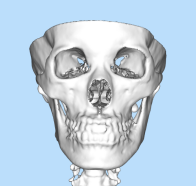 | 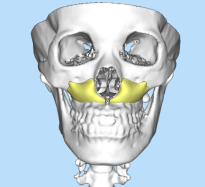 |
| 3 | 31 | Female | PARA I  INFRA U  INFRA I | PARA I  INFRA U  INFRA I | 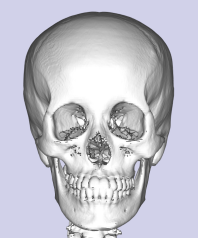 | 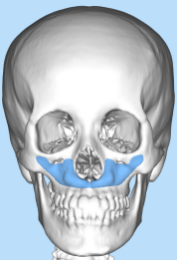 |
| 4 | 22 | Female | PARA U  PARA I  INFRA I | PARA U  PARA I  INFRA I | 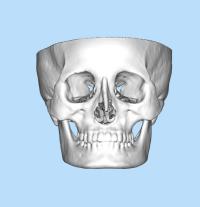 | 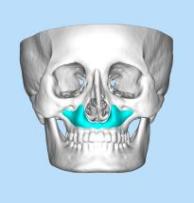 |
| 5 | 25 | Female | PARA U  PARA I  INFRA U  INFRA I  MALAR | PARA U  PARA I  INFRA I | 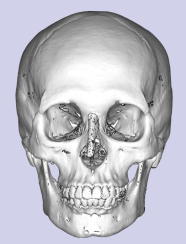 | 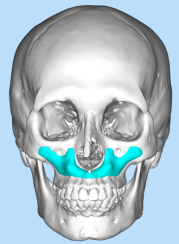 |
| 6 | 26 | Male | PARA U  PARA I  INFRA U  INFRA I | PARA U  PARA I  INFRA I | 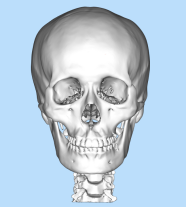 | 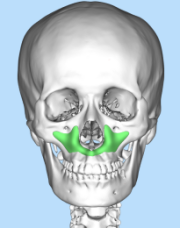 |
| 7 | 45 | Female | PARA I  INFRA U  INFRA I  MALAR | PARA I  INFRA U  INFRA I  MALAR | 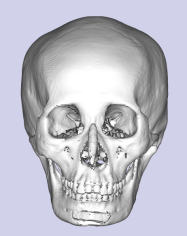 | 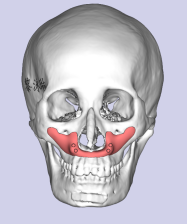 |
| 8 | 29 | Female | PARA U  PARA I  INFRA U  INFRA I  MALAR | PARA U  PARA I  INFRA U  INFRA I  MALAR | 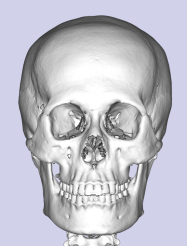 | 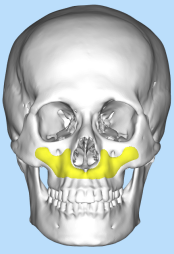 |
| 9 | 36 | Female | PARA I  INFRA I  MALAR | PARA I  INFRA I  MALAR | 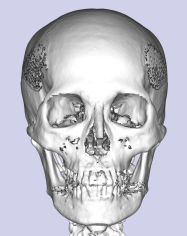 | 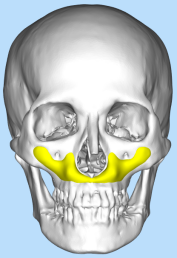 |
| 10 | 35 | Female | PARA I  INFRA U  INFRA I  MALAR | PARA I  INFRA U  MALAR | 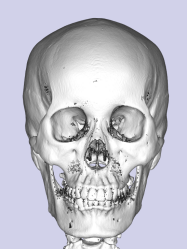 | 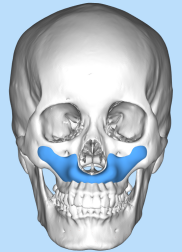 |
| 11 | 29 | Female | PARA U  PARA I  INFRA U  INFRA I  MALAR | PARA U  PARA I  INFRA I  MALAR | 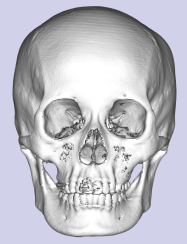 | 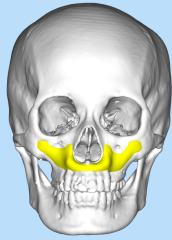 |
| 12 | 22 | Female | PARA I  INFRA U  INFRA I  MALAR | PARA I  INFRA I  MALAR | 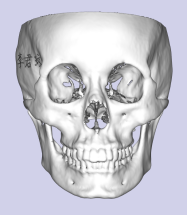 | 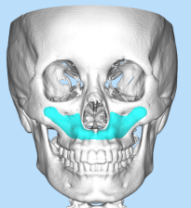 |
| 13 | 23 | Female | PARA U  PARA I  INFRA U  INFRA I  MALAR | PARA U  PARA I  INFRA I  MALAR | 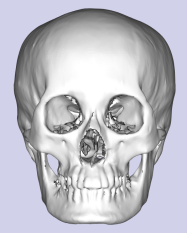 | 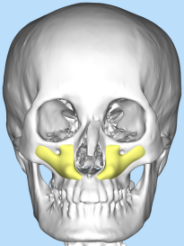 |
| 14 | 26 | Male | PARA U  PARA I  INFRA U  INFRA I  MALAR | PARA U  PARA I  INFRA I  MALAR | 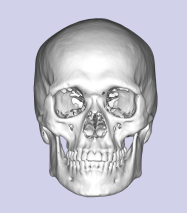 | 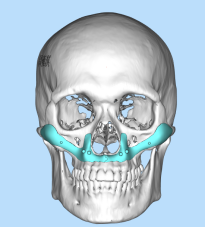 |
| 15 | 27 | Female | PARA U  PARA I  INFRA U  INFRA I  MALAR | PARA U  PARA I  INFRA U  INFRA I  MALAR | 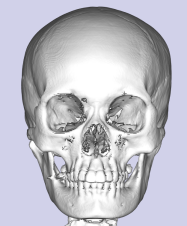 | 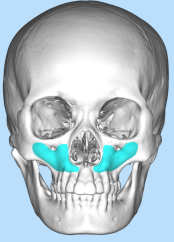 |
| 16 | 39 | Female | PARA I  INFRA U  INFRA I  MALAR | PARA I  INFRA U  INFRA I  MALAR | 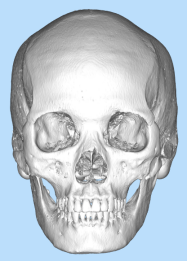 | 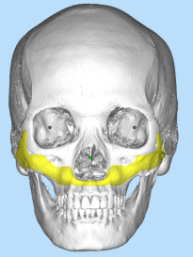 |
| 17 | 21 | Male | PARA U  PARA I  INFRA U  INFRA I  MALAR | PARA U  PARA I  INFRA U  INFRA I | 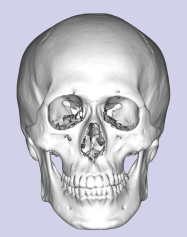 | 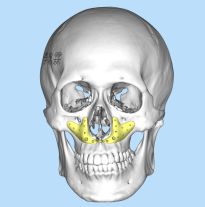 |
| 18 | 23 | Female | PARA U  PARA I  INFRA I  MALAR | PARA U  PARA I  INFRA I  MALAR | 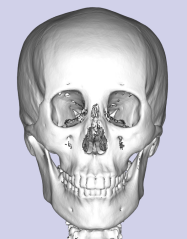 | 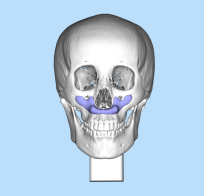 |
| 19 | 34 | Male | PARA I  INFRA U  INFRA I  MALAR | PARA I  INFRA I  MALAR | 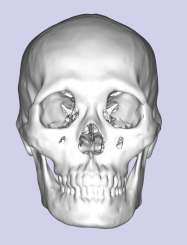 | 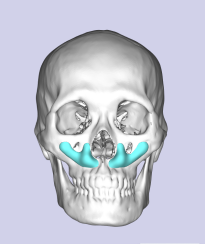 |
| 20 | 30 | Female | PARA U  PARA I  INFRA U  INFRA I  MALAR | PARA U  PARA I  INFRA U  INFRA I | 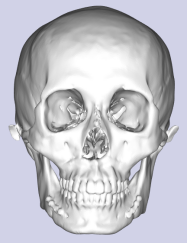 | 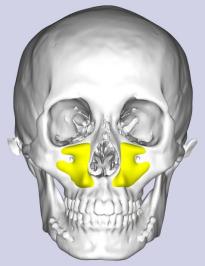 |
| 21 | 23 | Female | PARA I  INFRA I  MALAR | PARA I  INFRA I | 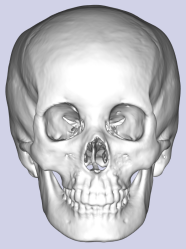 | 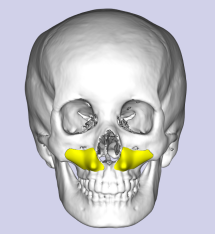 |
| 22 | 27 | Female | PARA U  PARA I  INFRA I  MALAR | PARA U  PARA I | 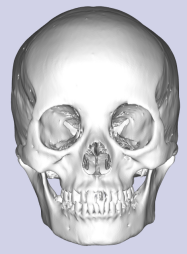 | 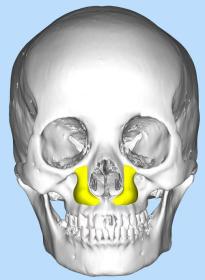 |
| 23 | 32 | Female | PARA I  INFRA U  INFRA I  MALAR | PARA I  INFRA I  MALAR | 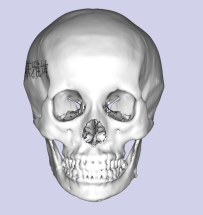 | 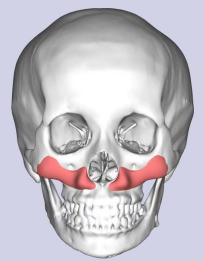 |
| 24 | 34 | Female | PARA I  INFRA U  INFRA I  MALAR | PARA I  INFRA U  INFRA I  MALAR | 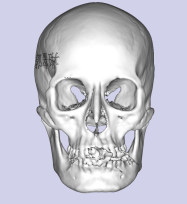 | 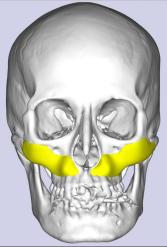 |
| 25 | 30 | Male | INFRA U  INFRA I  MALAR  SUBMALAR | INFRA U  INFRA I  MALAR | 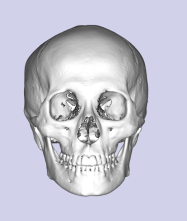 | 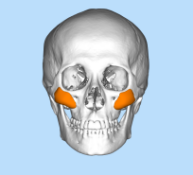 |
| 26 | 50 | Female | PARA I  INFRA I | PARA I  INFRA I | 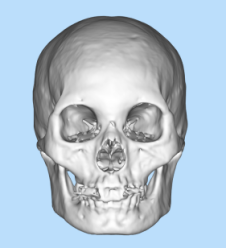 | 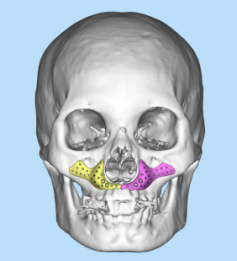 |
| 27 | 24 | Female | PARA U  PARA I | PARA U  PARA I | 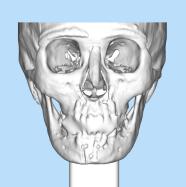 | 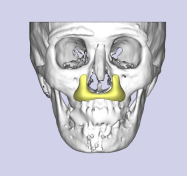 |
| 28 | 22 | Male | PARA U  PARA I | PARA U  PARA I | 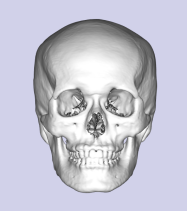 | 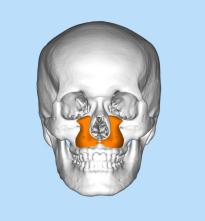 |
| 29 | 39 | Female | PARA U  PARA I  INFRA U  INFRA I  MALAR | PARA U  PARA I  INFRA I  MALAR | 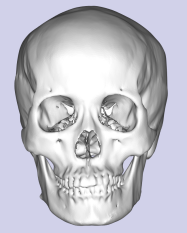 | 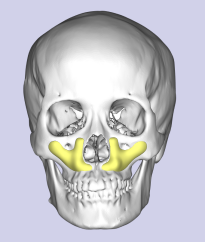 |
| 30 | 26 | Female | PARA I  INFRA U  INFRA I  MALAR | PARA I  INFRA U  INFRA I  MALAR | 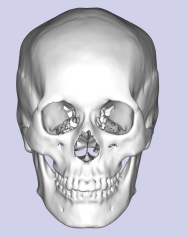 | 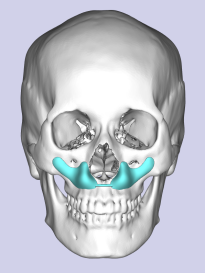 |
| 31 | 24 | Female | PARA U  PARA I  INFRA U  INFRA I  MALAR | PARA U  PARA I  INFRA I | 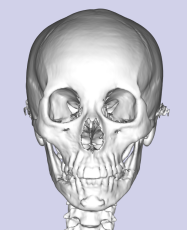 | 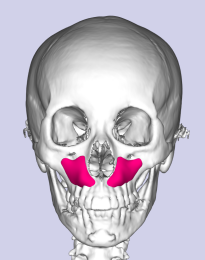 |

PARA U, upper paranasal region; PARA I, lower paranasal region; INFRA U, superior infraorbital region; INFRA I, inferior infraorbital region; MALAR, zygomatic/malar region; SUBMALAR, subzygomatic/submalar region.
